# A Non-Invasive Medical Device for Parkinson’s Patients with Episodes of Freezing of Gait †

**DOI:** 10.3390/s19030737

**Published:** 2019-02-12

**Authors:** Catalina Punin, Boris Barzallo, Roger Clotet, Alexander Bermeo, Marco Bravo, Juan Pablo Bermeo, Carlos Llumiguano

**Affiliations:** 1 Telecommunications Research Group, Universidad Politécnica Salesiana, Cuenca 010105, Ecuador; bbarzallo@ups.edu.ec (B.B.); abermeo@ups.edu.ec (A.B.); mbravog@ups.edu.ec (M.B.); jbermeo@ups.edu.ec (J.P.B.); 2 Networks and Applied Telematics Group, Universidad Simón Bolívar, Caracas 89000, Venezuela; clotet@usb.ve; 3 Neurology department, Hospital Vozandes Quito, Quito 170521, Ecuador; carlos.llumiguano@yahoo.com

**Keywords:** sensors, neurodegenerative disorders, clinical assessment, Parkinson’s disease, freezing of gait, discrete wavelet transform, vibratory stimulus

## Abstract

A critical symptom of Parkinson’s disease (PD) is the occurrence of Freezing of Gait (FOG), an episodic disorder that causes frequent falls and consequential injuries in PD patients. There are various auditory, visual, tactile, and other types of stimulation interventions that can be used to induce PD patients to escape FOG episodes. In this article, we describe a low cost wearable system for non-invasive gait monitoring and external delivery of superficial vibratory stimulation to the lower extremities triggered by FOG episodes. The intended purpose is to reduce the duration of the FOG episode, thus allowing prompt resumption of gait to prevent major injuries. The system, based on an Android mobile application, uses a tri-axial accelerometer device for gait data acquisition. Gathered data is processed via a discrete wavelet transform-based algorithm that precisely detects FOG episodes in real time. Detection activates external vibratory stimulation of the legs to reduce FOG time. The integration of detection and stimulation in one low cost device is the chief novel contribution of this work. We present analyses of sensitivity, specificity and effectiveness of the proposed system to validate its usefulness.

## 1. Introduction

Parkinson’s disease (PD) is a neurodegenerative chronic illness that affects movement, making it difficult for patients to comfortably perform tasks of everyday life such as: walking, stair climbing, writing, eating, etc. The World Health Organization rates PD as the second most common neurodegenerative disorder. According to the Parkinson’s Disease foundation, approximately 10 million people suffer from this disease worldwide [1,2]. People with PD experience both motor and non-motor symptoms. Typical motor symptoms during the early stages of PD are resting tremors, rigidity, and bradykinesia.

PD imposes a chronic burden not only on the patients but also on their personal and social environments. The difficulty to control movement produced by PD has a negative impact on the social and psychological behavior of the patient, who feels isolated and useless to perform common and simple tasks. Other important motor symptoms present during the disease’s middle stage include cramping (dystonia), dyskinesia, loss of postural reflexes, and Freezing of Gait (FOG). FOG is a major motor symptom of PD that shows up during the advanced stages of the disease. It is characterized by a brief episode of involuntary absence of locomotion, i.e., a sensation of being stuck in place, which is experienced by the patient especially when trying to initiate a step or when navigating through or turning around obstacles. FOG episodes cause serious difficulties in mobility and balance that significantly increase the risk of falling, thereby potentially producing serious injury, including bone fracture. Most PD patients affected by FOG usually are aged between 60 and 80 years. Therefore, if injured, these patients require permanent assistance and care by relatives as well as by specialized healthcare personnel.

Being a disease presently without a cure, treatments for PD are aimed at suppressing or ameliorating specific PD symptoms. Currently used symptom treatment types include pharmacological (medication), invasive (e.g., surgical, deep brain stimulation), and non-invasive and minimally invasive (e.g., transcranial and transcutaneous stimulation) interventions. Other more general treatment types consist of lifestyle modifications such as diet and exercise.

The use of non-invasive sensors is an effective approach for monitoring [3] gait and detecting motor symptoms such as FOG. Non-invasive sensors used for this purpose can be classified as either portable or stationary. Portable sensors may be attached to clothing [4,5] or to adequate supports [6,7,8,9,10,11,12]. They have the advantage of not limiting the PD patient’s travel space. Stationary (a.k.a. environmental) sensors are distributed at fixed positions throughout the personal environmental space of the PD patient [13] allowing the acquisition of a wider range of characteristics.

Both types can be used for the detection and prediction of FOG episodes, depending on the type of processing their output data is subjected to. Processing tools and techniques used for this purpose include: Power Spectral Density (PSD) [5], root mean square error (RMSE) [6], Fast Fourier Transform (FTT) [7,9], Artificial Intelligence (AI) [10,12,14] and Discrete Wavelet Transform (DWT) [14]. The use of some of these processing tools within common and easily accessible technologies, such as the mobile smartphones, already has achieved sensitivities and specificities greater than 70% [7,9,10,13].

Symptom detection sensors and data processing algorithms are used, in conjunction with external (non-invasive) sensory stimulation, for treatment of FOG. Among the most significant sensory type of external stimulation interventions are the auditory [7,11], visual [15] and tactile [4,6,10] modalities. Although early auditory or vibratory stimulation per se are not known to prevent FOG [16,17], they are nonetheless able to reduce the length of FOG episodes [6,11,16,18]. With this in mind, we present and describe here a novel low cost, compact, comfortable and integrated (detection + stimulation) real-time system intended to induce prompt resumption of gait during FOG episodes. The proposed integrated system consists of two light-weight devices, which are attached to the lower limbs of the PD patient, strategically placed to avoid discomfort. The devices sense gait and send the resulting data to be processed through a DWT-based Java encoded algorithm, designed to detect FOG episodes, in a mobile Android application. As soon as the FOG episode is detected, the system generates and applies a vibratory stimulation to help the PD patient to quickly regain gait, thus reducing the probability of serious injury.

## 2. Parkinson’s Disease

**Figure 1:**
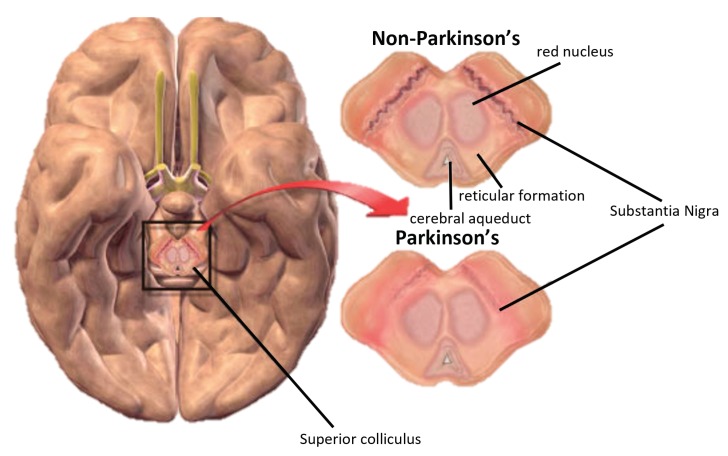
How Parkinson’s disease originates [25].

PD is a multi-systemic neurodegenerative disorder that affects the human nervous system, specifically the dopamine-producing (“dopaminergic”) neurons in the *substantia nigra* region of the brain. Dopamine is essential for sending messages to control and coordinate movement [19]. It acts as a messenger between the *substantia nigra* and the *striatum*, an area of the brain responsible for controlled smooth movement [19,20], as shown in Figure 1.

As was already mentioned, the most characteristic motor symptoms of the disease are resting tremor, limb rigidity, bradykinesia, and postural instability [1,2]. The diagnosis of PD depends on the presence of one or more of these four motor symptoms, as well as on the presence of other motor and non-motor secondary symptoms [5,21], such as changes in writing (micrography), reduction of facial expression and loosing of arm swinging and gait [22], constipation, olfactory dysfunction, psychiatric symptoms (such as apathy, anxiety, depression, dementia and psychosis), sleep disturbances, hypophonia, drooling (due to reduced swallowing) and pain [20,23,24].

Early studies about PD were mostly aimed at describing movement and motor disorders and at differentiating the stages of the disease. The degree of PD can be estimated using a widely accepted metric, the unified scale for the evaluation of Parkinson’s disease (UPDRS) [26]. The UPDRS value lays in the range from 0 to 176, where 0 represents the healthy condition and 176 represents total disability condition. This scale is based on the following three factors:

1. Mood, mental and behavioral,
2. Activities of daily living,
3. Motricity factor, which ranges from 0 (symptom-free condition) to 108 (severe motor condition) [27].

Symptoms and signs of PD vary from person to person, often beginning on one side of the body and usually continuing to worsen on that side, as symptoms begin to affect both sides. Evolution may be slow in some patients while it can be quicker in others.

Drugs used to reduce motor symptoms in PD can cause neuropsychiatric disorders. Among them, dopaminergic receptor agonists are the most frequently produced. However, there are no well-designed comparison studies about the frequency of such disorders in relation to the type of treatment used [28,29].

PD symptoms, including the FOG episodes, progressively worsen over time [30,31]. There are monitoring devices, based on accelerometers and gyrometers, which can be placed on different parts of the body to detect FOG episodes. Although these devices cannot prevent by themselves the occurrence of FoG, they can be used to trigger stimulation mechanisms to induce the resumption of gait [30].

### 2.1. Symptoms of Parkinson’s Disease

PD symptoms may be separated into three categories: primary motor symptoms, secondary motor symptoms, and pre-motor symptoms. They all progressively worsen as the disease advances. Table 1 lists the three categories.

Gait disorders caused by PD, such as FOG, have important effects on the health of the patient, most notably the risk of falling, which can cause injuries with serious consequences. Falls cause stress, pain, and are the leading cause of death from injuries in the elderly. In fact, more than a third of PD patients older than 65 suffer at least one fall per year, representing 65% of all their injuries. As a consequence, PD patients develop increasing fear of falling, which causes stress and produces a significant psychological impact on their lives [32].

### 2.2. **Freezing of Gait** ***(FOG)***

Movement freezing during the march is an episodic motor function disturbance, known as Freezing of Gait (FOG). FOG episodes last only a few seconds, and rarely exceed 30 s duration [33]. FOG is commonly observed in PD patients during the advanced stage of the disease [34,35]. Patients usually describe the episode as a feeling of having your feet “glued to the ground.”. FOG episodes may be triggered by different factors: attempting to start or continue the march, changing gait speed or direction of the march, presence of obstacles, walking in narrow spaces, monotone color environments, etc. Actual causes of FOG are still not well known, although there are some hypotheses, such as freezing being caused by the inability to generate a normal amplitude step length, or asymmetry of gait [36]. There does not seem to be a direct correlation between the frequency of FOG and other PD motor symptoms, such as stiffness and bradykinesia. However, FOG ocurrence is inversely proportional to the presence of tremors [37,38]. There is also evidence that indicates that L-Dopa and dopamine agonists contribute to the development of FOG. Neurodegeneration associated with normal aging seems to be a contributing factor [34,36]. The use of dopamine antagonists such as ropirinole [39] and pramipexole [40] can increase the frequency of freezing. FOG that occurs during the off phase of PD responds to L-Dopa, while freezing that occurs during the on phase does not [41]. Evidence indicates that L-Dopa or dopaminergic agonists can contribute to the development of freezing [38]. On phase patients do not respond to L-Dopa, suggesting possible involvement of non-dopaminergic pathways [41]. The inability to generate normal step length can trigger freezing [34]. Likewise, alteration in visual perception may be involved also in the genesis of FOG [39].

Appraisal of FOG is usually performed by a team of neuropsychiatric experts using certain tools and methods, such as: the Unified Scale for Parkinson’s disease (UPDRS) to determine the stage of the disease, the freezing of gait questionnaire (FOGQ) to determine presence of FOG [42]. They are complemented by an evaluation of the emotional and cognitive status, as well as the quality of life of the patient.

## 3. Mathematical Tool for Processing

### 3.1. Wavelet Theory

The wavelets operate analogously to Fourier analysis in some applications. The main difference that wavelets have with Fourier transforms is that wavelets perform local analysis, which makes them appropriate for the analysis of signals in the time-frequency domain, while Fourier transforms are global [43,44]. Wavelet techniques allow to divide a complex function into simpler ones and study them separately. They are appropriate for the analysis of image and biomedical signals, since they allow for decomposing a signal in subband, allowing the calculation of energy for each subband of decomposition.

The term “wavelet” is used to define the functions that are used to sample the signal (1):

(1) $$S\left(\tau ,\alpha \right)=\int_{-\infty }^{+\infty }S(t)\frac{1}{\sqrt[{}]{\alpha }}\Psi^{*}\left(\frac{t-\tau }{\alpha }\right).\mathrm{dt},$$

where $\Psi^{*}$ is the conjugate of the mother wavelet that will be scaled and run point by point to determine the levels of comparison with the signal S(t).

A wavelet function is a small wave, whose energy is concentrated in time and serves as a tool for the analysis of transient phenomena, non-stationary and variants in time [45]. In a wavelet mother, a signal S(t) can be decomposed into:

(2) $$\begin{array}{cc} S & =A1+D1\\ & =A2+D1+D1\\ & =A3+D3+D2+D1\\ & =\ldots \end{array}$$

**Figure 2:**
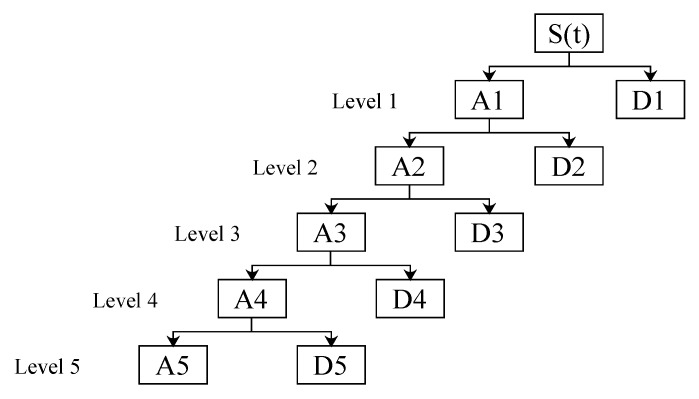
Decomposition wavelet in five levels: tree topology.

Thus,

$$=A_{j}+\sum_{j\leq J}D_{j},$$

where:

(3) $$A_{j}\left(t\right)=\sum_{k}c_{jk}\phi_{j}\left(t-k\right),$$

(4) $$D_{j}\left(t\right)=\sum_{k}d_{jk}\psi_{j}\left(t-k\right),$$

where $A_{j}$ and $D_{j}$ are the coefficients of approximation and detail respectively of the signal S(t) at the level *j* (see Figure 2); $\phi_{j}$ and $\psi_{j}$ are the scaling function and the wavelet function at level *j* for reconstruction; $c_{jk}$ and $d_{jk}$ , given by the wavelet transformed, there are coefficients of the function scaling and wavelet coefficients in the level *j* and the change of time *k*, respectively. The analysis wavelet allows the use of large intervals of time in those segments where greater accuracy is required at low frequency and smaller regions where high frequency information is required.

### 3.2. The Discrete Wavelet Transform

The Discrete Wavelet transform (DWT) is very similar to the discrete Fourier transform (DFT), but, instead of using sine and cosine functions like the latter, it uses a type of function called scale functions and wavelets. These functions combine the double characteristic of orthogonality (so that the reconstruction is the same as the transformation), as well as compact support in space [46].

The DWT of a function *f*(*x*) is given by the following expression (5):

(5) $${\mathrm{DWT}}_{\phi }f\left(j,\tau \right)=\int_{-\infty }^{+\infty }f\left(x\right)\phi_{j,\tau }^{*}\left(x\right)dx.$$

### 3.3. Energy of the Wavelet Coefficients

The energy in these components and their wave coefficients are related to the energy of the original signal. According to Parseval’s theorem, the energy contained in the signal is equal to the sum of the energy contained in the coefficients of detail and approximation in the different resolution levels of the wavelet transform [47,48]. That is, the signal energy can be decomposed in terms of the coefficients of transformation. Equations (6) and (7) express this theorem in function of different times (*k*) and scales (*j* = 1, …, *l*):

(6) $$EDC_{j}=\sum_{k=l}^{N}{\left|DC_{j,k}\right|}^{2},j=1,\ldots ,l,$$

(7) $$EAC_{l}=\sum_{k=l}^{N}{\left|AC_{j,k}\right|}^{2},$$

where *N* is the number of details coefficients $\left(DC_{j}\right)$ and approximation $\left(AC_{l}\right)$ in each decomposition level. In Equation (8), the total energy of the wavelet coefficients is detailed:

(8) $$E_{tot}=EAC_{l}+EDC_{j}.$$

## 4. Diagnostic Tests

In order to validate the system, the results were submitted to calculations of: sensitivity (9), specificity (10) and effectiveness (11), taking into account its calculation parameters: True Positive (*TP*), False Negative (*FN*), True Negative (*TN*) and False Positive (*FP*) [49]. The total duration of the signals that were processed and analyzed is 480 s of each patient (15 signals of 32 s for patient), doing a total of 3840 s for eight patients:

(9) $$Sensitivity=\frac{TP}{TP+FN}\times 100\%,$$

(10) $$Specificity=\frac{TN}{TN+FP}\times 100\%,$$

(11) $$Effectiveness=\frac{Resumption\;of\;the\;gait}{TP}\times 100\%.$$

### 4.1. Data Collection and Processing

The tests were performed in eight patients between 60 and 84 years of age, of which seven suffer from Parkinson’s disease (PD) and a healthy subject considered as the control patient. The characteristics of the patients are presented in Table 2, along with the identification of the degree of the disease and the episodes of Freezing of Gait (FOG) that occurred during the system test (described by a neurologist).

**Figure 3:**
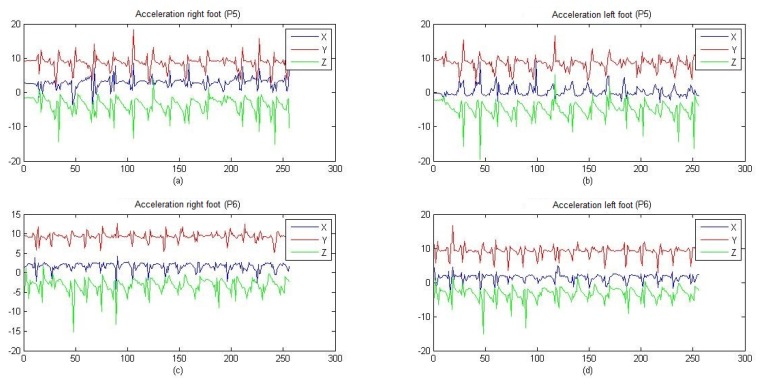
Acceleration in patients 5 and 6, performed in the lower extremities.

Figure 3 compares the similarity between both low extremities (right and left); through the application of cross-correlation, it was determined that both extremities present the same behavior, indicating that there is no need to acquire the signals of the two legs. In all patients, the acceleration of motor activity of the posterior sural nerve of the right leg was recorded and a superficial stimulation was performed on the intersection of the posterior tibial nerve and the lateral plantar nerve in both legs. A test circuit was established with the typical scenarios of the occurrence of an episode of freezing. For 32 s, the data of the walk was registered crossing the circuit of each patient, which in turn contain 256 values of the module of the tri-axial acceleration. During testing, patients made some activities to stimulate FOG occurrence:

- Walk in straight line,
- 180 degree turns on the walk,
- Climb steps.

The processing was done in the Arduino Pro Mini module (3.3 V/40 mA), based on the ATmega328 microcontroller (Atmel Corporation, San Jose, California, United States of America) that works on an open source platform, in which the rest of the modules and elements are connected. Acceleration data of the lower extremity is acquired by means of MPU 6050, where the Arduino Pro mini is in charge of applying a pre-processing and sending the measurements through bluetooth to the Smartphone, after the processing is forwarded a bit (1 or 0) for the Control of the motor, where the device replicates the bit when it is sent via radio frequency to the left device in order to control the second motor.

### 4.2. Hardware

**Figure 4:**
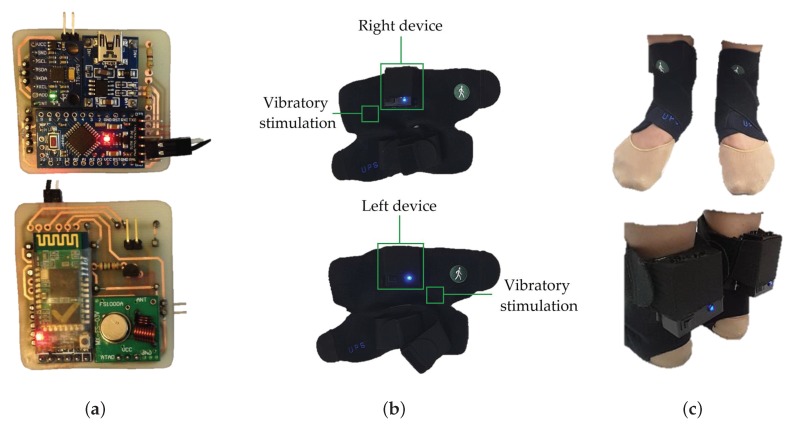
(**a**) Printed Circuit Board with components; (**b**) Encapsulated device in ergonomic support; (**c**) Device placed in patient.

We used two devices. On the right leg, one device acquires the data, sends it via Bluetooth to the Smartphone and executes the vibratory stimulation when necessary, and, on the left leg, another device only executes the vibratory stimulation. Both are built on a Printed Circuit Board (PCB) to double layer with a supply of individual energy by means of two batteries of lithium of 3.7 V/500 mA, in addition to a load system and covered with encapsulated of Poly-lactic Acid (PLA). The right device contains the following elements: Triaxial accelerometer (MPU 6050), Bluetooth Module v 2.0 (HC-05), Radio Frequency (RF) Emitter (433 MHz), On/OFF switch, LED indicator and vibratory motor; while the left device consists of: RF receiver (433 MHz), voltage amplifier module, On/OFF switch, LED indicator and vibratory motor (see Figure 4a).

The already encapsulated devices are located on two ergonomic supports and adjustable to the lower extremities, designed in polyamide and elastane material, as shown in Figure 4b. The devices and vibratory motors are placed in these supports, so that they coincide with the proposed location for the acquisition and stimulation. The total weight is 220 g for the right device and 210 for the left. In Figure 4c, it is possible to visualize the devices and the motor that remains fixed thanks to the adjustment of the supports, allowing complete mobility and the use of the patient’s usual footwear. In addition, the LED indicators, charging port and switches are displayed.

### 4.3. Software

Inside the Arduino, the acceleration of the sensor to 8 Hz (frequency of sampling) is acquired, it is transmitted by radio frequency and Bluetooth with the help of virtual libraries, and the pins of input and output of data are also configured. The data frame received in the Smartphone is decomposed to separate the values of the acceleration in each axis, and Equation (12) is applied to acceleration data, which determines the module resulting from the triaxial acceleration in the patient:

(12) $$Acceleration=\sqrt[{}]{AccX^{2}+AccY^{2}+AccZ^{2}}.$$

The result of the previous calculation is stored in a dynamic FIFO (First In First Out) vector of 256 elements. When this vector is full, with first 256 elements, DWT is executed. The DWT parameters used are: wavelet = Haar, scaling = 2, decomposition levels = 5 and filter order = 1. The algorithm is encoded in Java to run inside an Android platform developed application.

The saved signal is multiplied with a vector and its result is debugged by orthogonal low-pass and high-pass filters. This is sent to a second filtering that depends on the scale and length of the signal, and allows all data to be divided and stacked in frequency ranges, covering the size of the sampling frequency. The data groups resulting from the low-frequency filter (according to their level of decomposition) continue to be filtered, separated and grouped according to the frequency spectrum; the grouped vector compendium is designated as wavelet coefficients. The wavelets’ coefficients of all levels of decomposition correspond to the same number of elements that exist in the vector of the acquired signal, grouped in a new vector and separated as detail and approximation coefficients.

From the coefficients of the DWT, it is possible to estimate the total energy level of the signal as well as the amount of energy stored in each frequency subband that was established in the wavelet decomposition. Equations (6)–(8) establish the amount of energy in Joules by levels of the coefficients of detail, approximation and total, respectively.

The calculation of the DWT and wavelet energy is made for every eight new pieces of acceleration data, which, according to the sampling frequency is every 1 s, then the detection of FOG and therefore the decision to activate the stimulus is made every 1 s. The time of the stimulation will be until the energy levels exceed the proposed threshold.

### 4.4. Graphical User Interface

**Figure 5:**
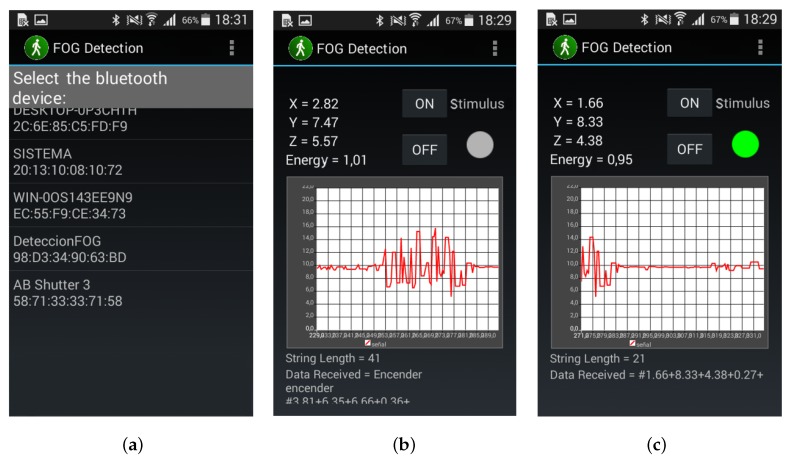
Graphical interface of application “FOG Detection” (**a**) home screen; (**b**) results screen/stimulus OFF; (**c**) results screen/stimulus ON.

An Android mobile application “*FOG Detection*” was developed for graphic interaction with the patient, and Figure 5a shows the first screen that appears when the application is opened. The following elements are found on this screen:

- List of previously linked bluetooth devices,
- MAC address of each physical bluetooth device.

Once a device to link from the list of available Bluetooth’s devices is selected, the screen changes to Figure 5b. This screen interface shows the following:

- Acceleration data in its three axes (*x*, *y*, *z*),
- Amount of energy in the signal,
- Buttons for manual activation and deactivation of the vibratory stimulus,
- Real time graph of triaxial acceleration,
- Indicator of motors activation.

In Figure 5c, active state of the automatic stimulation is observed, by means of a green indicator, representing the appearance of a FOG and the activation of vibratory stimulus.

## 5. Results and Discussion

From acquisition and processing were obtained results of acceleration and energy, respectively. The acceleration data provide characteristics of the walk and allow for differentiating and extracting characteristics of the episodes of FOG, while the energy levels establish the beginning, duration and end of the episodes, permitting the activation of the vibratory stimulation until the resumption of the gait. The patient’s response to stimulation during the walk is evaluated by calculations of sensitivity, specificity, and effectiveness, to quantify the ability of the system to differentiate between patients who have FOG from those who do not.

### 5.1. Acceleration

**Figure 6:**
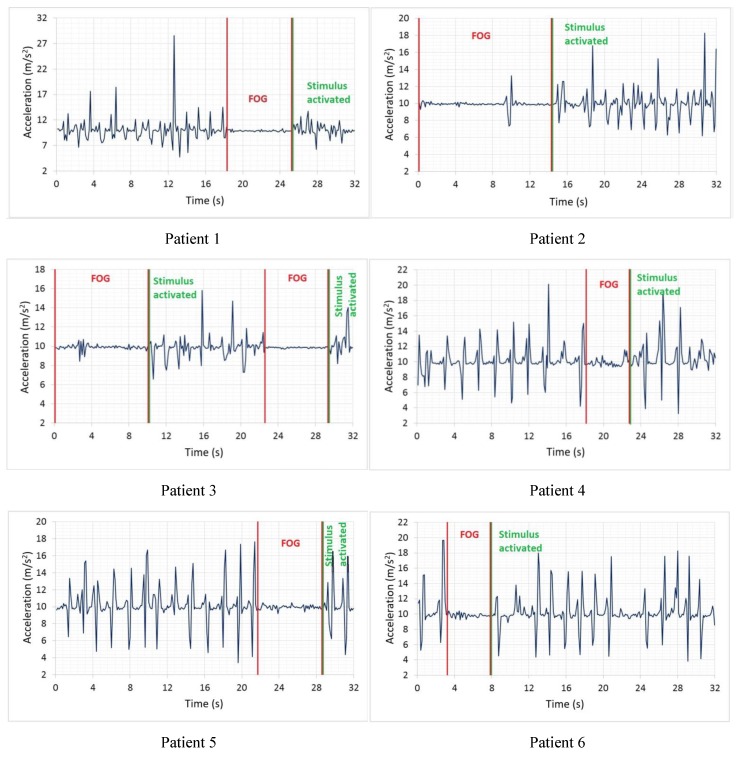
Signals of patients with FOG.

The triaxial acceleration data are received and correlatively stored in a file with “.csv” extension. Figure 6 show some graphs of recorded signals where patients 1 to 6 exhibit episodes of FOG, along with the beginning of the episode and the resumption of the walk, while patients 7 and 8 did not show episodes of FOG during testing.

The segment limited by parallel lines of red establishes the episodes of FOG that were diagnosed by the specialist. The green lines establish the beginning of the resumption of the gait in patients affected by the vibratory stimulus.

**Figure 7:**
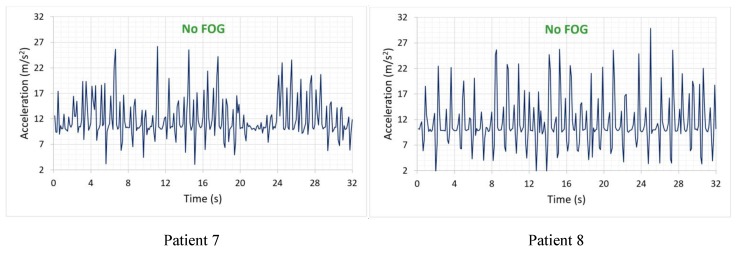
Signals of patients without FOG.

It is notable that signals with FOG (Figure 6) contain lower extremities accelerations that change less rapidly and have less amplitude compared to signals of Figure 7, which correspond to patients without PD. In turn, in Figure 6, there is a greater number of peaks and uniformity in their signals, while the signal segments that were diagnosed as FOG present small variations in peak-to-peak amplitude around 9.81 m/s${}^{2}$, which indicates a state of almost at rest of the lower extremities.

In Figure 6, FOG episodes occur at different instants and periods of duration with a range between 9 to 11 m/s${}^{2}$ of amplitude, establishing a peak-to-peak magnitude of approximately 2 m/s${}^{2}$, regardless of age and sex. Two episodes of FOG are presented in patient 3 (Figure 6), corresponding to 32 s duration of the accumulated signal, becoming an indicator that the patient may be in a grade 4 of the PD; patients with this degree of disease require long-term continuous stimuli to continue the gait. The above establishes that, depending on the degree of the disease, the duration of its episodes of FOG can increase.

Patient 7 (Figure 7) has the signal of a patient diagnosed with Parkinson’s disease without FOG in grade 1, with its mild symptoms and the disease is controlled by medication administration; there is a decrease in energy between 20 and 24 s of the signal due to an incomplete turn made by the patient. This isn’t a FOG episode. While patient 8 (Figure 7) is a healthy patient, within the same age range as previous patients, its acceleration is uniform and periodic, that is to say, it does not present flaws in the behavior of the walk.

### 5.2. Energy Levels

**Figure 8:**
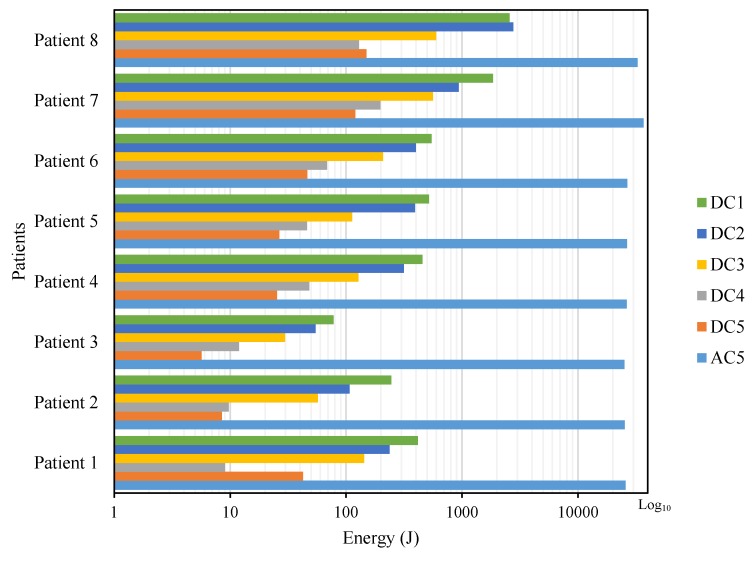
Distribution of total energy in the subbands of the wavelet coefficients.

Figure 8 shows the amount of energy that contains the wavelet coefficients, a product of the DWT processing. It shows the difference in energy levels between patients with episodes of FOG and those who do not have them. In addition, the distribution of the energy for each level of detail decomposition and approximation coefficients is observed.

In [50], it is established that the frequency of FOG occurs in the bandwidth of 3 to 8 Hz. This range was placed within the detail coefficients of the first level (DC1), excluding from the analysis the coefficients in the frequency range of 3 to 4 Hz because they do not provide relevant information in FOG detection, but it is necessary to consider all the coefficients for the calculation of the total energy and energy levels of the subbands, as included in the Equations (6)–(8). Based on the above considerations, for calculations, processing and analysis, there is a spectrum of frequencies for the presence of FOG from 4 to 8 Hz, whose frequency ranges are contained by detail coefficients from level 1 (DC1) to detail coefficients of level 5 (DC5).

**Figure 9:**
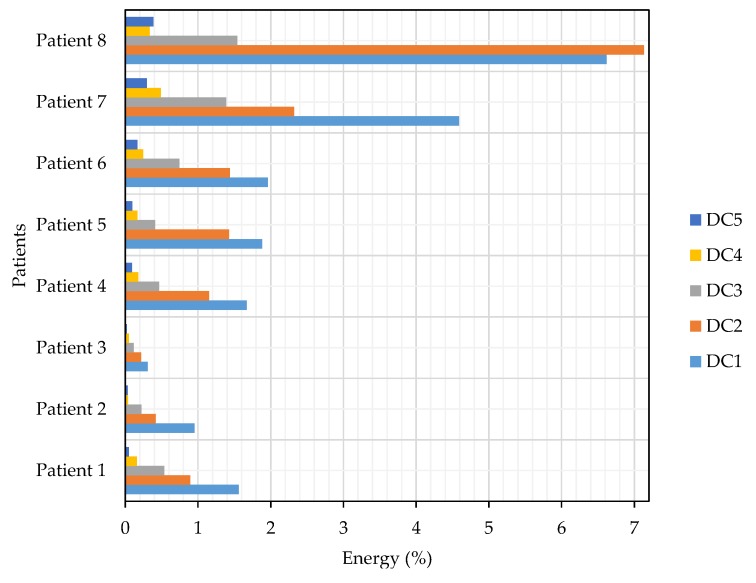
Percentage distribution of energy in the subbands of the coefficients.

According to the results of the tests in Figure 8, each patient, regardless of the degree of their illness, presents different amounts of energy because everyone maintains a different walking pattern, establishing that there are no significant characteristics that differentiate the presence of FOG in the signals. Then, it is necessary to do a percentage comparative analysis, except for AC5, but included in the calculation as shown in Equation (13), which is contrasted in Figure 9, which presents the percentages of energy from DC’s from level 1 to level 5 (five levels of wavelet decomposition) derived from eight signals from the eight patients described in Table 2: six with PD and FOG (Patients 1 to 6), 1 with the PD without FOG (Patient 7) and one without the PD or FOG (Patient 8):

(13) $$Energy=\frac{{\left|\mathrm{AC}5\right|}^{2}+{\left|{\mathrm{DC}}_{j}\right|}^{2}}{{\left|\mathrm{AC}5\right|}^{2}+\sum_{i=l}^{5}{\left|{\mathrm{DC}}_{i}\right|}^{2}}\times 100\%.$$

Based on the results in Figure 9, it is shown that the total energy of signals with FOG is lower than that of signals without FOG (see Figure 8), due to the scarce or null activity of movement in the lower extremities during a FOG episode. Because the FOG occurs in the DC1 frequency band, the DC1’s energy value is compared between all patients described in Figure 9, where the percentage of energy in patients 7 and 8 is higher than in patients 1 to 6, approximately over 4%, while patients who had FOG episodes have energy levels below 2%. The value of 2% is taken as the limit level for the activation of the proposed vibratory stimulation, which should increase with the resumption of activity in the lower extremities and thus avoid possible falls.

### 5.3. System Validation

Table 3 and Table 4 summarize the results of the tests performed with the system developed in conjunction with the neurosurgeon’s assessment. Taking into account the parameters useful for calculating sensitivity, the amount of TP and FN was recorded in Table 3, while the amount of TN and FP, necessary for the calculating specificity, is recorded in Table 4.

Using the values recorded in Table 3 and Table 4 in the Equations (9)–(11), it obtains a specificity of 86.66% and a sensitivity of 60.61% in the FOG detection, while the system’s effectiveness for the resumption of walking after the freezing is detected is 80%.

Table 5 highlights an improvement in the time reduction of the FOG episodes of each patient using vibrational stimulation versus measurements without any stimulation, approximately 27% reduction in the duration of FOG episodes.

To verify system usability, all patients were asked about system comfort and stimulus effect, with two questions:

- Have you ever experieced any discomfort while wearing the system?
- Do you feel uncomfortable from any aspect with system feedback (vibratory stimulus)?

All of them manifest that wearing the system does not represent any discomfort, and system feedback is soft enough to not be annoying, but detectable enough to help.

To detect FOG, many techniques were tested by different researchers. Table 6 shows the most recent papers about it. Most of them combine Video Recording and Acceleration [51,52,53,54,55,56]; Acceleration alone was used by [57,58,59]; Acceleration in combination with angular velocity by [60] and in combination with Inertial Measurement Unit sensor by [61,62]; Video Recording alone by [63]; Using Microelectromechanical systems by [64] and using Electroencephalography by [14,65]. The main objective of our research is to find an efficient system to detect and to stimulate with an affordable cost based on motor frequency analysis that can be improved with the implementation of neural networks and hip acceleration measures, in addition to exploring vibratory stimulation as a blockage of FOG. It has a similar performance in specificity and a lower average performance of 22.96% in sensitivity with respect to the other investigations, but our system works in real time (some studies use external hardware to processes data offline with, of course, better results), it is low cost, compact, comfortable and integrated (detection + stimulation).

## 6. Conclusions

The motor problems of Parkinson’s disease originate in the brain and branch out through the nervous system, leading to changes in gait such as FOG. The use of an exogenous stimulus allows the brain to break from the freezing state of the lower extremities and resume the gait.

This device integrates a wireless, low cost, compact, comfortable, easy to use and portable system designed, developed and implemented for patients with Parkinson’s disease who have episodes of FOG.

This device integrates an algorithm that detects in real time and accurately episodes of FOG and then stimulates the patient to resumption of gait.

The duration of the FOG episodes is variable for each patient as well as the reaction time of the patient to the vibratory stimulus; although its appearance is strongly linked to situations of stress and low self-esteem, such as the difficulty of crossing narrow places or climbing steps. During system tests, it was observed that the resumption of the gait presented sudden and accelerated movements compared to normal walking.

The use of this developed device helps the patient to move and perform their daily activities without restrictions, and, at the same time, allows their real-time monitoring. In addition, storage of gait data that would help to understand, process and clarify FOG episodes occurs.

The mathematical tool of the DWT is useful to find differences in the acquired signals and to establish thresholds variables that define an episode of FOG, with the possibility of characterizing other types of motor anomalies.

Future work with more patients and with different processing techniques, as, for example, neural networks, will be done to improve specificity and sensitivity, maintaining the restriction of a low cost system. This would provide the possibility of differentiating an episode of FOG from a voluntary pause in gait, correcting system levels of sensitivity, and calibrating effectiveness of the stimulus.

## Figures and Tables

**Table 1:** Motor and non-motor symptoms of Parkinson’s disease.

Primary Motor	Secondary Motors	Pre-Motor
Tremor, is presented in the first signs of PD.	The freezing of gait, important signal of the PD that he does not explain by the rigidity or bradykinesia.	Loss of the sense of smell.
Bradykinesia, slow movement indicating reduction of spontaneous movement.	Micrograph, contraction of the patient’s fist.	Constipation, sleep disorder.
Rigidity, inflexibility in the extremities, neck and trunk.	Lack of facial expression	Mood Disorder.
Postural instability, a tendency to be unstable when placed vertically.	Festivity, uncontrollable acceleration in the march	Low blood pressure when standing. Orthostatic hypotension

**Table 2:** Characteristics of the patients evaluated.

	Age	Gender	Parkinson’s Disease	Degree of PD		Freezing of Gait Episodes
				On	Off	
Patient 1	78	Female	Yes	4	3	Yes
Patient 2	84	Male	Yes	4	3	Yes
Patient 3	69	Male	Yes	3	2	Yes
Patient 4	67	Female	Yes	3	1	Yes
Patient 5	71	Female	Yes	4	2	Yes
Patient 6	73	Male	Yes	3	1	Yes
Patient 7	62	Female	Yes	2	1	No
Patient 8	60	Male	No	0	0	No

**Table 3:** Results and resumption of gait for patients with episodes of FOG.

	FOG Episodes Diagnosed by the Neurosurgeon	FOG Episodes Detected by the Proposed System		Resumption of Gait through Vibratory Stimulus
		True Positive (TP)	False Negative (FN)	
Patient 1	6	4	2	4
Patient 2	2	2	1	2
Patient 3	5	2	2	3
Patient 4	3	2	2	1
Patient 5	4	3	3	3
Patient 6	7	4	2	3
**Total**	27	20	13	16

**Table 4:** Results of patients who do not have episodes of FOG.

		System Results	
	Signals Analyzed	True Negative (TN)	False Positive (FP)
Patient 7	15	14	1
Patient 8	15	12	3
**Total**	30	26	4

**Table 5:** FOG time with stimulation and without stimulation.

	Time Average without Stimulation [s]	Time Average with Stimulation [s]	Improvement Percentage [%]
Patient 1	7.74	4.52	41.63
Patient 2	9.87	9.22	6.59
Patient 3	10.19	6.83	33.02
Patient 4	7.04	5.92	15.87
Patient 5	7.18	4.97	30.84
Patient 6	4.56	3.06	32.89
Total average	7.76	5.75	26.81

**Table 6:** Comparison of methodologies and results of work oriented to the detection of FOG.

Reference	Methodology			Results	
	Number of Patients/Episodes	Acquisition	Processing	Specificity (%)	Sensitivity (%)
[14]	5	EEG* (cortical regions: frontal, central, parietal)	Time-frequency analysis with combinations of DWT* and SVM*	89.5	83.1
[51]	20/98	VR* and Acc* (hip)	Step rate, freezing and energy index	84.1	70.1
[52]	20	VR* and Acc* (hip)	Support vector machines, stride detection, spectral power and motor status threshold	94	96
[53]	15	VR* and Acc* (hip)	Support vector machines	>90	>90
[54]	10/237	VR* and Acc* (ankle, thigh and lower back)	Continuous wavelet transform	81.01	84.9
[55]	18	VR* and Acc* (hip)	Diffuse Logic: Freezing index, derived energy ratio, variation of the cadence and power spectrum	>86	>78
[56]	18/>200	Visual, motion and depth	Support Vector Machines and Logistic Regression classifier.	91	91
[57]	10/237	Acc* (ankle, thigh and lower back)	Power spectrum, Freezing index, FFT*	81.6	73.1
[58]	8/237	Acc* (ankle)	Classifier: Freezing index, energy, FFT* and statistical characteristics	85	70
[59]	10/237	3 × Acc*	DL* (Convolutional Neural Networks)	90.6	69.29
[60]	15/46	Acc* and angular velocity (hip)	Automatic learning algorithm	91.7	86
[61]	32	IMU* sensor, Acc* of Smartphone (hip)	Variations of K during threshold crossings	93.41	97.57
[62]	21	IMU* sensor, (Acc*, gyroscope and magne-tometer)	Data representation + DL* (Convolutional Neural Networks)	89.5	91.9
[63]	30/25	VR*	Time-frequency analysis with combinations of FFT* and WT*	>95	75–83
[64]	7	MEMS* (headset or shins)	Dynamic Time Warping and ANN*	96.7	94.5
[65]	6	EEG* (cortical regions: Frontal F4)	Short time Fourier Transform	88	84.2

* EEG = Electroencephalography, DWT = Discrete Wavelet Transform, SVM = Support Vector Machine, VR = Video recording, Acc = Acceleration, FFT = Fast Fourier Transform, MEMS = Microelectromechanical systems, ANN = Artificial Neural Network, IMU = Inertial Measurement Unit, WT = Wavelet Transform, DL = Deep Learning.
